# Spermine Alleviates Chondrocyte Senescence and Extracellular Matrix Degradation by Suppressing NF-κB Signaling, with Odc1 Acting as a Potential Upstream Regulator

**DOI:** 10.3390/ijms27219859

**Published:** 2026-11-03

**Authors:** Rui Shi, Rongling Feng, Junming Zhu, Panghu Zhou

**Affiliations:** Renmin Hospital of Wuhan University, 99 Zhangzhidong Road, Wuchang District, Wuhan 430060, China; 2024283020159@whu.edu.cn (R.S.); 2019305231073@whu.edu.cn (R.F.); 2025183029018@whu.edu.cn (J.Z.)

**Keywords:** osteoarthritis, chondrocyte senescence, extracellular matrix, spermine, ornithine decarboxylase 1, nuclear factor-κB signaling pathway

## Abstract

Osteoarthritis (OA) is a prevalent degenerative joint disease with no disease-modifying therapy currently available. Spermine (SPM), a natural polyamine, shows therapeutic potential, but its role and mechanism in OA remain unclear. In this study, SPM expression was examined in OA cartilage, and the effects of exogenous SPM on chondrocyte senescence and extracellular matrix degradation were assessed in vitro and in a rat OA model. Bioinformatics analysis and Odc1 knockdown experiments were performed to identify the upstream regulator of SPM. The results showed that SPM was significantly downregulated in TBHP-stimulated chondrocytes. Exogenous SPM supplementation alleviated chondrocyte senescence, inhibited matrix degradation, and attenuated cartilage degeneration in vivo. Mechanistically, SPM inhibited NF-κB signaling, and Odc1 was identified as a key upstream regulator of SPM. In conclusion, SPM alleviates chondrocyte senescence and matrix degradation in vitro and attenuates OA progression in vivo. These effects are partially achieved by suppressing NF-κB signaling and upregulating Odc1, indicating that SPM is a promising therapeutic candidate for OA.

## 1. Introduction

Osteoarthritis (OA) is a chronic degenerative joint disorder characterized by progressive cartilage degradation, osteophyte formation, and subchondral bone sclerosis [1]. Epidemiological studies indicate that the global age-standardized prevalence of OA increased from 4.71% in 1990 to 6.85% in 2019, with the number of affected individuals increasing by 113.25% over this period [2]. Driven by accelerating global population aging and persistently rising obesity rates, OA incidence and related disability are projected to keep increasing through 2050, posing a mounting public health challenge [3]. Current therapeutic strategies for OA are broadly categorized into surgical and non-surgical approaches. Surgical interventions, such as joint replacement, are generally reserved for patients with late-stage disease and thus provide limited benefits for early-stage intervention. In contrast, non-surgical treatments including physical therapy, non-steroidal anti- inflammatory drugs (NSAIDs), and glucosamine remain widely used [4]; however, these interventions have proven insufficiently effective at halting or reversing the underlying pathological progression of OA. Moreover, long-term use of these agents is associated with a range of adverse effects, including gastrointestinal toxicity, hepatotoxicity, and anaphylactic reactions [5]. Therefore, a thorough understanding of OA pathogenesis is critical to lay the foundation for developing novel mechanism-based molecular therapeutics.

The progression of OA is influenced by multiple factors, including age, gender, obesity, previous joint injuries, mechanical overload, genetic susceptibility, and inflammatory diseases [6]. Chondrocyte senescence and disruption of joint homeostasis are recognized as pivotal regulatory elements involved in OA advancement [7]. As the unique cellular component of articular cartilage, chondrocytes play a vital role in maintaining extracellular matrix (ECM) homeostasis [8]. Nevertheless, age-induced chondrocyte senescence impairs the ability of chondrocytes to sustain tissue stability [9]. Senescent chondrocytes release a range of senescence-associated secretory phenotype (SASP) factors, such as pro-inflammatory cytokines, matrix-degrading enzymes, and growth factors [10]. These SASP factors mediate chronic inflammatory responses and accelerate the degradation of core ECM components, including type II collagen and aggrecan [11], ultimately disrupting cartilage structural integrity and disturbing joint homeostasis during OA pathogenesis. Notably, a significant accumulation of senescent cells has been documented in both articular cartilage and synovial tissues from OA patients, with their abundance positively correlated with disease severity [12]. Moreover, pharmacological elimination of senescent cells has been shown to retard OA progression and create a microenvironment conducive to cartilage regeneration [13].

Spermine (SPM) is a natural polyamine widely found in eukaryotic cells, with multiple biological functions including promoting cell proliferation and differentiation, mediating immune regulation, maintaining mitochondrial homeostasis, and enhancing autophagy [14]. SPM can be synthesized endogenously or obtained from exogenous sources such as diet [15]. Existing studies have confirmed that abnormal SPM metabolism is closely associated with multiple diseases. Notably, exogenous SPM supplementation has been proven to effectively delay and even ameliorate various age-related disorders, including cardiovascular disease, neurodegenerative disease, and metabolic disorders [16]. Furthermore, recent research has shown that OA patients have a lower serum SPM-to-spermidine ratio than healthy individuals [17], suggesting that SPM may exert a protective effect in OA.

Ornithine decarboxylase 1 (*Odc1*) is the first rate-limiting enzyme in the polyamine biosynthesis pathway, catalyzing the decarboxylation of ornithine into putrescine—the essential precursor of spermidine and SPM [18]. Recent studies have demonstrated that *Odc1* knockdown accelerates cellular senescence and upregulates the expression of senescence-associated factors in bronchial epithelial cells [19], but its regulatory function in chondrocytes has not been clarified. Accordingly, in this study, we systematically screened SPM metabolism-related genes and, through a series of experiments, further verified the critical regulatory role of *Odc1* in OA. Based on these findings, we established a TBHP-induced in vitro OA model to explore the regulatory mechanism of the *Odc1*–SPM–NF-κB axis in OA so as to provide new molecular targets for OA intervention.

## 2. Results

### 2.1. SPM Network Pharmacological Analysis and Its Downregulation in OA

**Figure 1:**
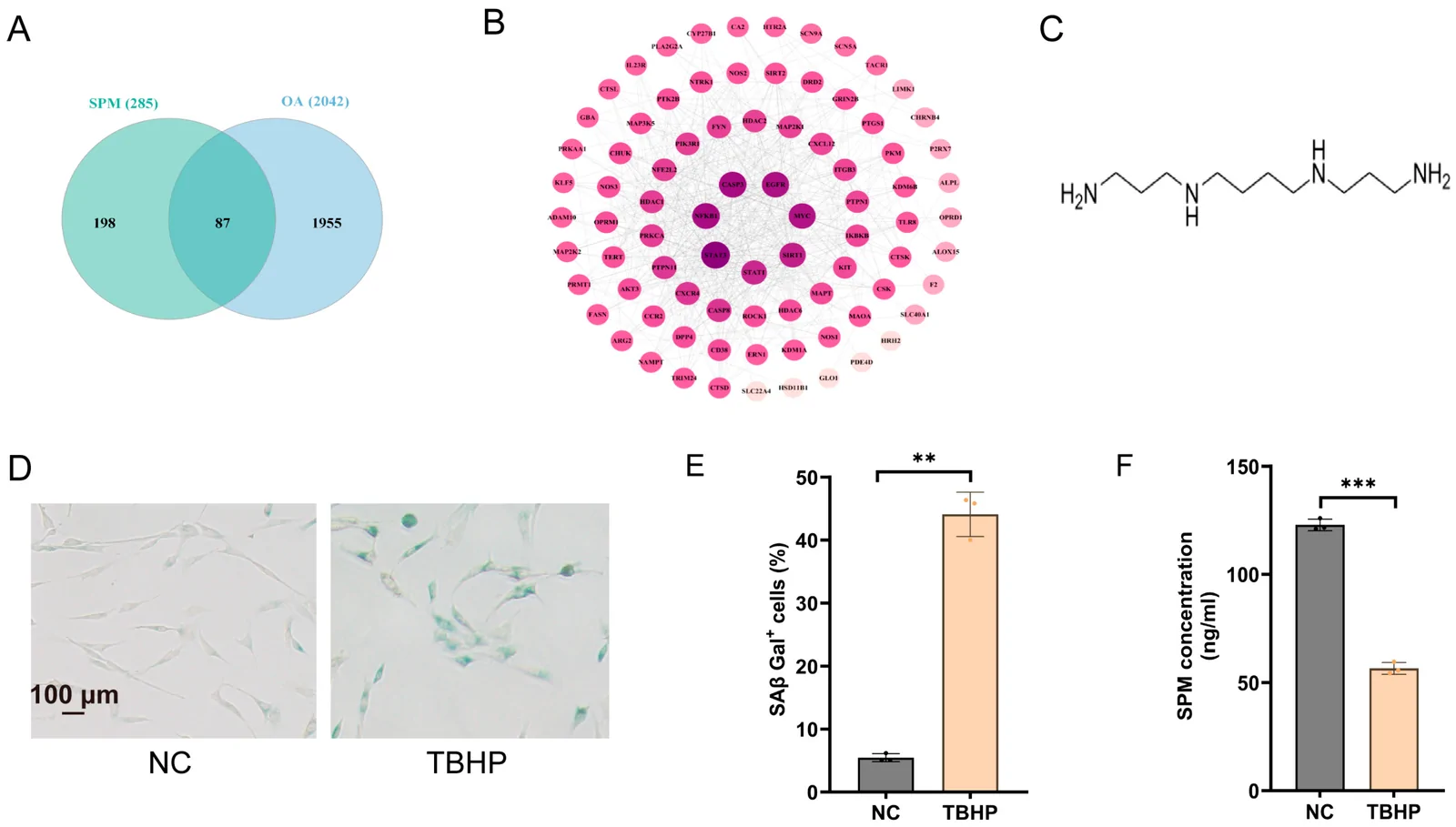
Network pharmacology-based prediction of SPM targets and validation of its downregulation in OA: (**A**) Venn diagram showing overlapping targets between SPM-associated genes and OA-related disease genes. (**B**) PPI network of the intersecting targets. (**C**) Chemical structure of SPM. (**D**,**E**) Representative images and quantitative analysis of SA-β-Gal staining in TBHP-induced chondrocytes (scale bar: 100 μm); blue-stained cells indicate senescent chondrocytes. (**F**) Relative SPM levels in chondrocytes determined by ELISA (*n* = 3). Data are presented as mean ± SD. ** *p* < 0.01; *** *p* < 0.001 vs. the NC group.

To predict the potential molecular targets of SPM in OA, a network pharmacology approach was initially employed. By leveraging the Swiss Target Prediction and PubChem databases, 285 SPM-related targets were identified, while 2042 OA-associated disease targets were retrieved from the GeneCards and OMIM databases. Venn diagram analysis uncovered 87 overlapping targets between these datasets (Figure 1A). The target genes were uploaded to the STRING database to construct a PPI network comprising 83 nodes and 538 edges, visualized using Cytoscape software. Based on degree value rankings, key genes such as NFKB1, SIRT1, EGFR, and STAT3 were identified as hub genes within the PPI network (Figure 1B), suggesting that these proteins may serve as critical hubs in suppressing OA progression.

Building on these findings, the study utilized tert-butyl hydroperoxide (TBHP) at a concentration of 40 μM to induce chondrocyte senescence over a 24 h period, thereby establishing a robust in vitro model [20]. This model was subsequently validated using SA-β-gal staining (Figure 1D,E). The chemical structure of SPM is shown in Figure 1C. Based on this model, changes in SPM expression under OA conditions were assessed using an ELISA kit. The results showed that SPM concentration was significantly decreased in the TBHP-treated group relative to the control group (Figure 1F), indicating that SPM deficiency may be involved in the pathological progression of OA.

### 2.2. SPM Alleviates Chondrocyte Senescence and ECM Degradation

**Figure 2:**
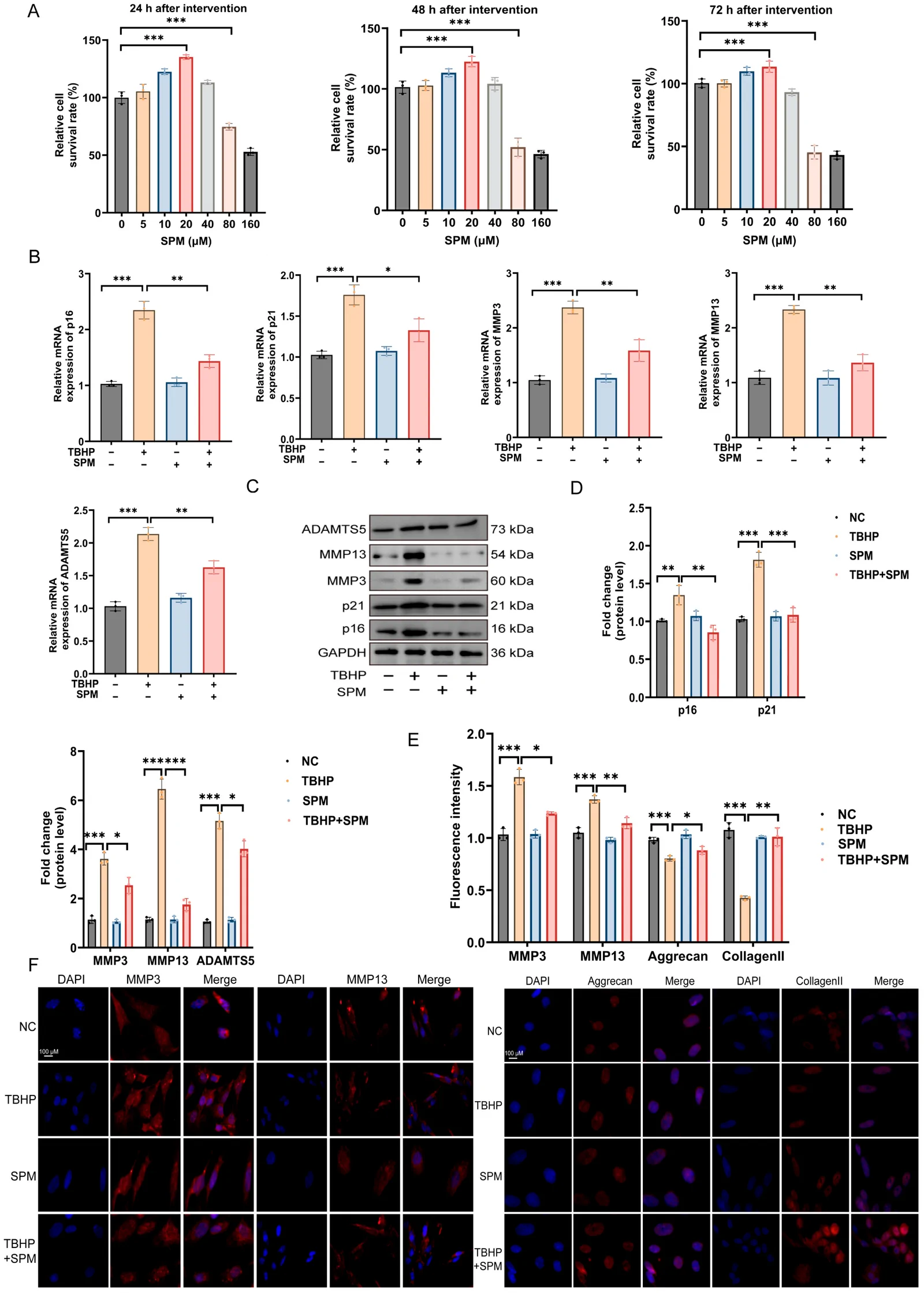
The impact of SPM on chondrocyte viability, senescence, and matrix metabolism: (**A**) Chondrocyte viability was assessed via the CCK-8 assay following exposure to SPM at concentrations spanning 0 to 160 µM for 24, 48, and 72 h. (**B**) RT-qPCR analysis determined the mRNA expression levels of senescence markers (*p16*, *p21*) and matrix-degrading enzymes (*MMP3*, *MMP13*, and *ADAMTS5*) in TBHP-induced chondrocytes treated with 20 µM SPM. (**C**,**D**) The corresponding protein expression levels were analyzed via Western blot, accompanied by semi-quantitative assessment. (**E**,**F**) IF staining demonstrated the influence of SPM on the expression of MMP3, MMP13, aggrecan, and type II collagen in TBHP-induced chondrocytes, complemented by semi-quantitative analysis (scale bar: 100 μm). Data are presented as mean ± SD (*n* = 3). * *p* < 0.05; ** *p* < 0.01; *** *p* < 0.001 vs. the TBHP group.

Given the marked reduction in SPM levels in the in vitro OA model, we next assessed the effect of exogenous SPM on chondrocyte viability. To establish a safe and effective concentration range, chondrocytes were exposed to varying concentrations of SPM (0–160 µM), and cell viability was evaluated using the CCK-8 assay. The results showed that SPM at concentrations of 5–20 µM sustained cell viability at all time points (24, 48, and 72 h). In contrast, concentrations above 40 µM markedly diminished viability, revealing a cytotoxic effect at elevated doses (Figure 2A). To further evaluate the dose-dependent anti-senescence effects of SPM, we performed SA-β-gal staining in TBHP-stimulated chondrocytes treated with increasing concentrations of SPM. The percentage of SA-β-gal-positive cells decreased progressively as SPM concentrations increased, reaching the lowest level at 20 µM (Figure S1). However, a slight rebound was observed at 40 µM, consistent with the reduced cell viability at this concentration in the CCK-8 assay (Figure 2A). Based on these findings, a concentration of 20 µM was chosen for subsequent mechanistic investigations. Subsequently, we explored the effects of SPM on chondrocyte senescence and ECM metabolism. RT-qPCR and Western blot analyses disclosed that TBHP stimulation notably upregulated the mRNA and protein expression of senescence markers (p16 and p21) as well as matrix-degrading enzymes (MMP3, MMP13, and ADAMTS5) (Figure 2B–D). Remarkably, co-treatment with SPM effectively counteracted these alterations. IF staining further validated that SPM inhibited the TBHP-induced upregulation of MMP3 and MMP13 while concurrently enhancing the expression of aggrecan and type II collagen (Figure 2E,F). In conclusion, these findings illustrate that SPM mitigates chondrocyte senescence and ECM degradation, thereby exerting a chondroprotective effect.

### 2.3. SPM Suppresses NF-κB Signaling Pathway Activation

**Figure 3:**
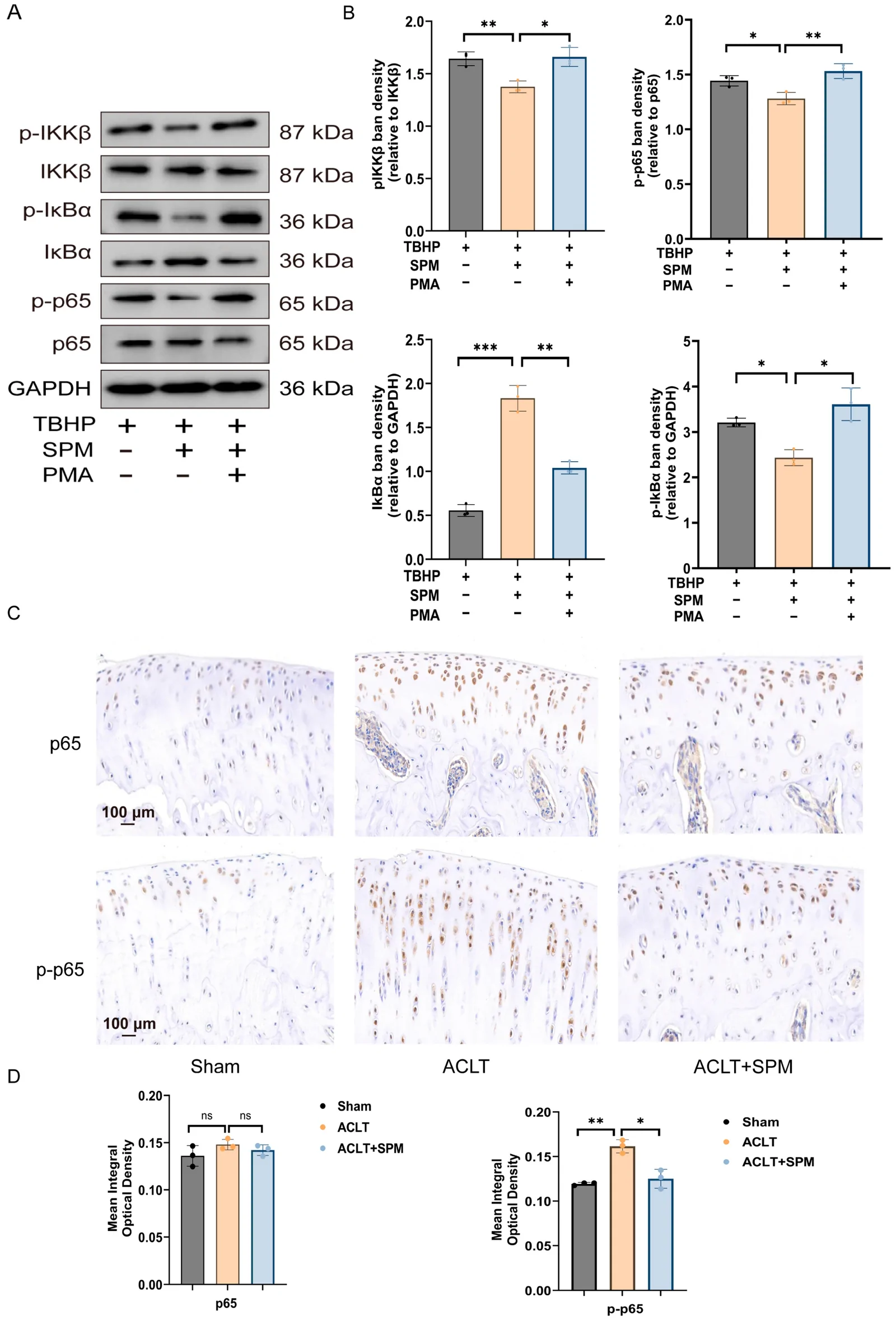
SPM suppresses TBHP-induced NF-κB signaling pathway activation: (**A**,**B**) Representative Western blots and densitometric quantification of p-IKKβ, IKKβ, p-IκBα, IκBα, p-p65, and p65 in chondrocytes (*n* = 3). (**C**,**D**) IHC staining and quantitative analysis of p-p65 and p65 expression in cartilage tissues. Data are presented as mean ± SD. * *p* < 0.05; ** *p* < 0.01; *** *p* < 0.001 vs. the TBHP group.

The NF-κB pathway plays a pivotal role in regulating various pathophysiological processes in OA, including cartilage matrix remodeling, chondrocyte apoptosis, synovial inflammation, and terminal chondrocyte differentiation [21]. Given its central importance and insights from PPI analysis, we investigated whether SPM mediates its protective effects in OA by modulating this pathway. Western blot analysis revealed that SPM treatment significantly attenuated TBHP-induced phosphorylation of IKKβ, IκBα, and p65, while concurrently preventing IκBα degradation. Notably, the addition of Phorbol 12-myristate 13-acetate (PMA) (20 ng/mL), a potent NF-κB activator [22], reversed these inhibitory effects, leading to a rebound in phosphorylation levels (Figure 3A,B). Together, these results indicate that SPM effectively suppresses NF-κB signaling. IHC staining further corroborated that SPM markedly reduced ACLT-induced p-p65 expression and p65 nuclear translocation (Figure 3C,D).

### 2.4. Odc1 Is Downregulated in OA

**Figure 4:**
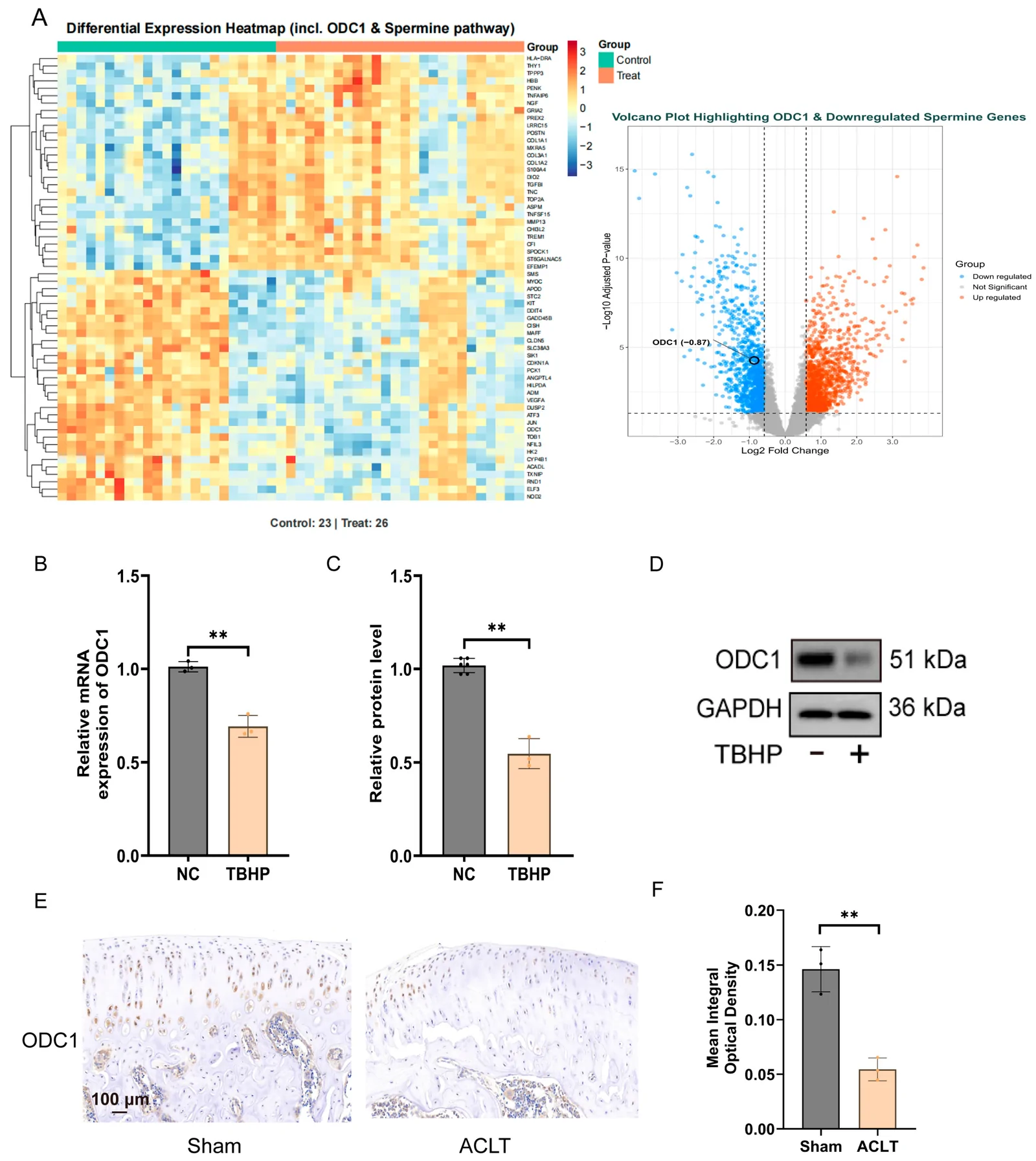
*Odc1* expression is downregulated in OA: (**A**) Volcano plot showing differentially expressed genes (DEGs) between OA (*n* = 26) and control (*n* = 23) cartilage samples from the GSE114007 and GSE169077 datasets. DEGs were defined as |log_2_(fold change)| > 0.5 and adjusted *p*-value < 0.05 (Benjamini–Hochberg correction). Red and blue dots represent up- and downregulated genes, respectively; grey dots indicate non-significant genes. The dashed vertical lines represent |log_2_FC| = 0.5, and the dashed horizontal line represents −log_10_(adj. *p* = 0.05). *Odc1* is specifically labeled. (**B**–**D**) RT-qPCR and Western blot analyses confirming decreased *Odc1* mRNA and *Odc1* protein levels in the rat OA chondrocyte model. (**E**,**F**) Representative IHC staining images and semi-quantitative analysis of ODC1 expression in OA cartilage tissues (scale bar: 100 μm). Data are presented as mean ± SD (*n* = 3). ** *p* < 0.01 vs. the NC group.

To identify the upstream regulator responsible for aberrant SPM metabolism, we first performed bioinformatics analysis, which revealed a marked downregulation of ODC1 in human OA cartilage tissues (Figure 4A). This observation was validated using an in vitro rat OA chondrocyte model, where RT-qPCR and Western blot analyses consistently verified the downregulation of *Odc1* at both transcriptional and translational levels (Figure 4B–D). Moreover, IHC staining and semi-quantitative analysis further validated the downregulation of ODC1 in OA (Figure 4E,F).

### 2.5. Knockdown of Odc1 Accelerates Chondrocyte Senescence and ECM Degradation

**Figure 5:**
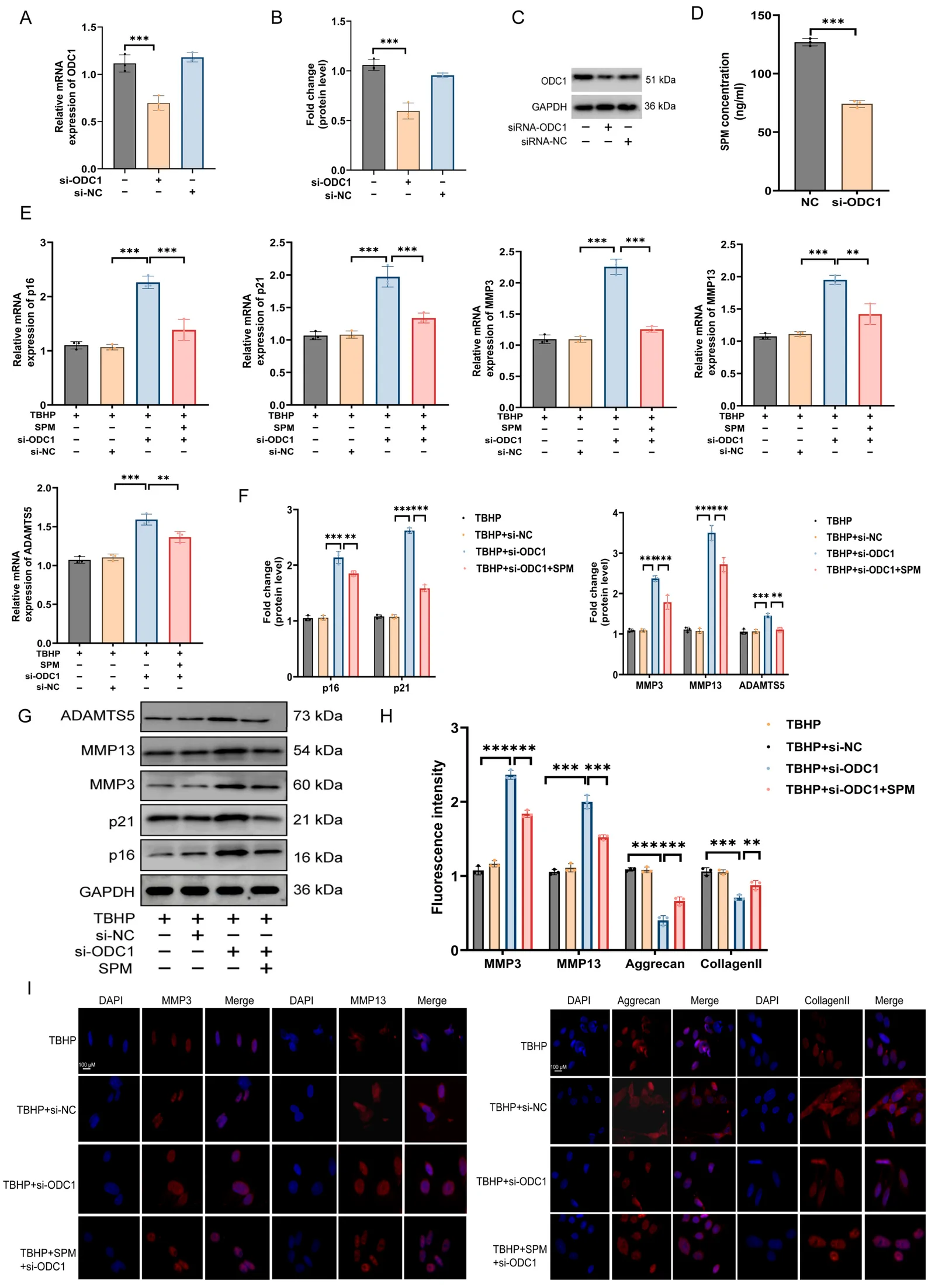
*Odc1* is associated with SPM synthesis and exerts chondroprotective effects against cellular senescence and extracellular matrix degradation: (**A**–**C**) The efficiency of *Odc1* knockdown was validated by RT-qPCR and Western blot analysis in chondrocytes transfected with *Odc1*-specific siRNA (si-*Odc1*) or a negative control siRNA (si-NC). (**D**) ELISA results demonstrated that silencing *Odc1* significantly reduced SPM levels. (**E**–**G**) qPCR and Western blot analyses revealed the effects of *Odc1* knockdown, with or without SPM supplementation, on the TBHP-induced expression of senescence markers (p16 and p21) and matrix-degrading enzymes (MMP3, MMP13, and ADAMTS5) in the following groups: TBHP, TBHP+si-NC, TBHP + si-*Odc1*, and TBHP + si-*Odc1* + SPM. (**H**,**I**) Representative IF images and semi-quantitative analysis illustrated the expression of MMP3, MMP13, aggrecan, and type II collagen in chondrocytes following *Odc1* knockdown with or without SPM supplementation after TBHP stimulation (scale bar: 100 μm). Data are presented as mean ± SD (*n* = 3). ** *p* < 0.01; *** *p* < 0.001 vs. the TBHP + si-*Odc1* group.

To determine whether SPM functions through *Odc1*, we knocked down Odc1 using a specific siRNA (si-Odc1), with a negative-control siRNA (si-NC) serving as the transfection control. Knockdown efficiency was confirmed by qPCR and Western blot, which showed that si-NC did not affect Odc1 expression compared with untransfected cells (Figure 5A–C). ELISA results showed that *Odc1* silencing significantly reduced SPM levels (Figure 5D), which suggests that *Odc1* may regulate SPM synthesis.

To further verify whether the chondroprotective effect of *Odc1* is mediated by SPM, we performed a rescue experiment by supplementing exogenous SPM in *Odc1*-knockdown cells. RT-qPCR and Western blot analyses revealed that *Odc1* knockdown significantly upregulated the mRNA and protein levels of senescence markers (p16 and p21) and matrix-degrading enzymes (MMP3, MMP13, and ADAMTS5) compared with the TBHP-stimulated untransfected control group. Noticeably, these effects were effectively reversed by co-treatment with SPM (Figure 5E–G). IF staining further confirmed that SPM supplementation in *Odc1*-knockdown cells suppressed TBHP-induced upregulation of MMP3 and MMP13 while promoting the expression of aggrecan and type II collagen (Figure 5H,I). Overall, these findings indicate that *Odc1* knockdown significantly exacerbates chondrocyte inflammation and ECM degradation, while SPM supplementation successfully rescues the pathological effects of *Odc1* deficiency. This further supports the conclusion that *Odc1* exerts its chondroprotective role primarily through the regulation of SPM synthesis.

### 2.6. SPM Alleviates Chondrocyte Senescence and Retards OA Progression in a Rat Model

**Figure 6:**
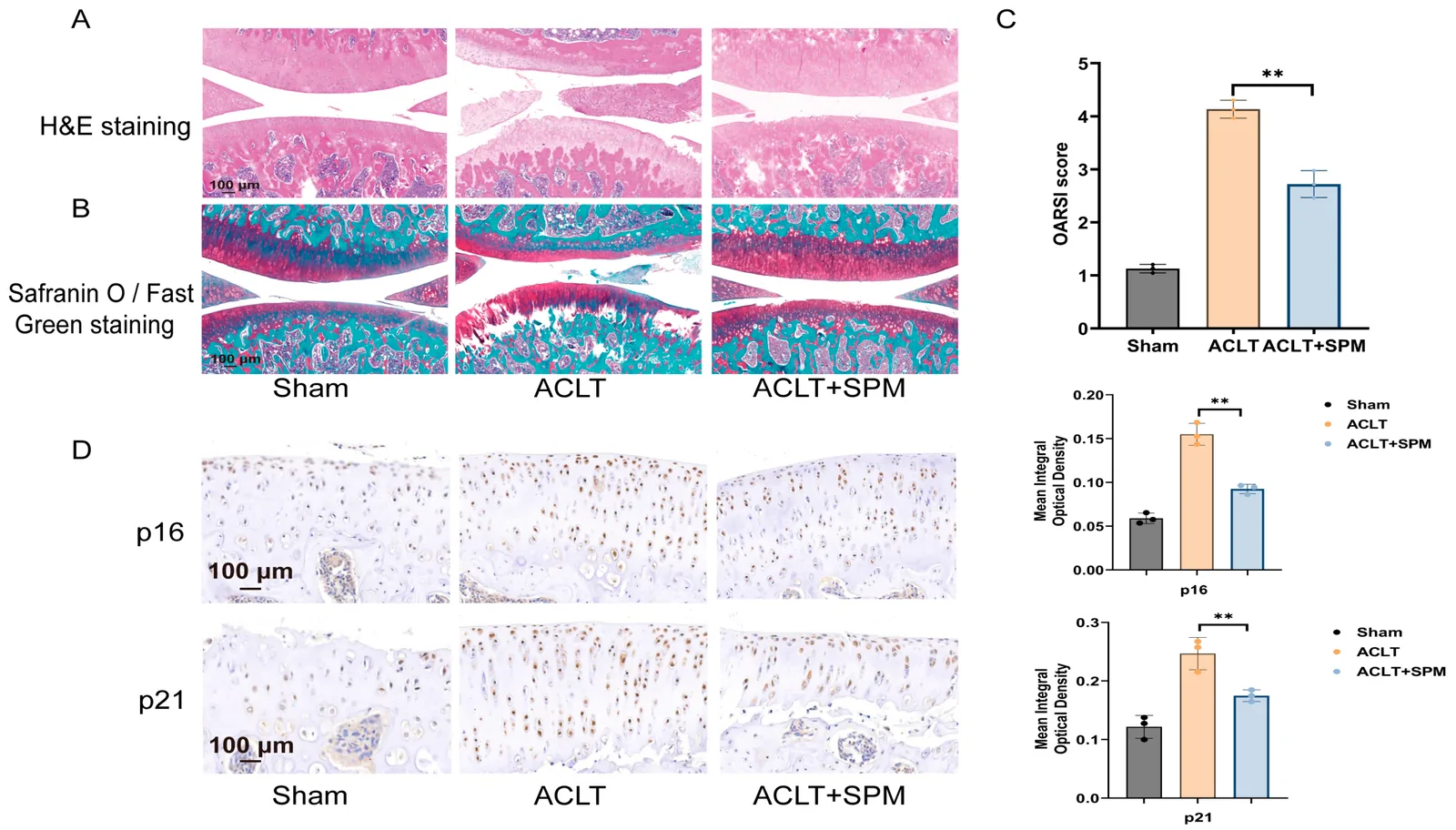
SPM alleviates ACLT-induced cartilage degeneration and chondrocyte senescence in a rat OA model: (**A**,**B**) Representative images from H&E and Safranin O-Fast Green staining depict knee joint sections of the Sham, ACLT, and ACLT + SPM groups (scale bar: 100 μm). (**C**) OARSI scores for cartilage damage are presented for each group. (**D**) IHC staining and semi-quantitative analysis reveal senescence markers p16 and p21 in cartilage tissues (scale bar: 100 μm). Data are presented as mean ± SD (*n* = 5 per group). ** *p* < 0.01 vs. the ACLT group.

To evaluate the structural impact of SPM on OA progression, we established a rat OA model via anterior cruciate ligament transection (ACLT). Coronal sections obtained at 8 weeks post-surgery revealed striking histological disparities among the groups. H&E staining demonstrated that the sham-operated group maintained a smooth and intact cartilage surface with uniformly arranged chondrocyte layers. In contrast, the ACLT group displayed severe cartilage erosion, surface irregularities, and a drastic decline in chondrocyte density. SPM treatment partially preserved the cartilage structure and significantly mitigated surface disruption (Figure 6A). Safranin O-Fast Green staining was employed to evaluate proteoglycan content within the cartilage matrix. The ACLT group displayed a substantial loss of Safranin O staining, indicating extensive proteoglycan depletion, while SPM administration significantly enhanced staining intensity, suggesting restoration of proteoglycan content (Figure 6B). OARSI histopathological scoring further confirmed that ACLT-induced cartilage damage was substantially ameliorated by SPM treatment (Figure 6C). IHC staining and semi-quantitative analysis revealed that SPM intervention potently reduced the expression of senescence markers p16 and p21 compared with the ACLT group (Figure 6D). Collectively, these findings demonstrate that intra-articular SPM administration effectively mitigates ACLT-induced cartilage degeneration, preserves proteoglycan content, and maintains joint structural integrity, underscoring its promising potential as a therapeutic agent for OA.

## 3. Discussion

Polyamine metabolism, which encompasses putrescine, spermidine, and SPM, has recently emerged as a research hotspot across multiple fields, including cancer, inflammation, autoimmunity, and degenerative diseases [23]. In cartilage biology, dysregulated polyamine metabolism is closely associated with the pathological progression of OA, particularly in the regulation of chondrocyte terminal differentiation and autophagy [24]. Among all polyamines, SPM has drawn special attention because its depletion is consistently observed in OA, highlighting the clinical importance of maintaining SPM homeostasis for this disease. Given its well-documented biological functions, it is reasonable to infer that SPM deficiency impairs the homeostatic capacity of chondrocytes and thus promotes OA progression.

In this study, we demonstrated that SPM is markedly downregulated in TBHP-stimulated chondrocytes. Exogenous SPM supplementation significantly reduced the expression of senescence markers in chondrocytes. Furthermore, SPM effectively reversed the upregulation of MMP3, MMP13, and ADAMTS5, while simultaneously promoting ECM synthesis. Considering the central role of NF-κB in mediating inflammation and matrix degradation in OA [25], we next explored whether SPM exerts its protective effects through this pathway. Our results showed that SPM alleviates OA progression by suppressing NF-κB activation and blocking p65 nuclear translocation. Notably, the NF-κB activator PMA reversed these inhibitory effects, further confirming that suppression of NF-κB signaling is a core mechanism underlying the anti-inflammatory and anti-senescence activities of SPM.

The therapeutic potential of SPM was further validated in vivo. In an ACLT-induced rat OA model, intra-articular injection of SPM substantially ameliorated cartilage erosion, restored proteoglycan content, lowered OARSI histopathological scores, and suppressed the expression of senescence markers p16 and p21. These results are consistent with our in vitro findings and confirm that SPM can maintain the structural integrity of joints in surgically induced OA models. However, the mechanism regulating SPM biosynthesis in chondrocytes remains unclear.

To address this gap, we next investigated the upstream regulation of endogenous SPM synthesis. *Odc1* is the rate-limiting enzyme of the polyamine biosynthesis pathway [18]. To explore whether *Odc1* controls endogenous SPM production in OA, we knocked down *Odc1* in primary chondrocytes with specific siRNA and found that this markedly impaired SPM synthesis and consequently attenuated its anti-senescence and matrix-protective effects. Notably, exogenous SPM supplementation effectively rescued these defects, indicating that the chondroprotective effects of *Odc1* are largely dependent on SPM production. Collectively, these findings demonstrate that SPM acts as a key downstream effector mediating the chondroprotective effects of *Odc1*. Since *Odc1* acts upstream of SPM in the polyamine biosynthetic pathway, restoring either *Odc1* expression or SPM levels may be an effective strategy for maintaining polyamine homeostasis and counteracting chondrocyte senescence in OA.

Despite these promising results, several limitations need to be acknowledged. First, although *Odc1* serves as the rate-limiting enzyme of the polyamine biosynthetic pathway, its knockdown theoretically depletes the entire polyamine pool—including putrescine, spermidine, and SPM—rather than SPM alone. However, which specific polyamine is depleted to a greater extent in chondrocytes remains to be determined due to the complexity of the polyamine metabolic network and its feedback regulation. Future studies should therefore focus not only on individual polyamines but also on their interactions and metabolic balance in OA. Second, our in vitro experiments only used TBHP stimulation, while OA pathogenesis involves a complex interplay of multiple factors, including mechanical stress and metabolic dysregulation. As a result, the representativeness of this single-factor model is inherently limited. Future studies should incorporate more comprehensive in vitro models, such as mechanical loading and co-culture systems, to better recapitulate the multifactorial pathogenesis of OA. Third, the ACLT-induced OA model primarily mimics post-traumatic OA and cannot fully reflect the pathological features of age-related or metabolic OA. Therefore, the long-term efficacy and safety of SPM need to be evaluated in more OA models with extended observation periods. Furthermore, all animals used in the in vivo experiments were male. Given the well-recognized sex differences in OA prevalence, severity, and molecular mechanisms, whether the chondroprotective effects of SPM observed in the present study are sex-dependent remains to be determined. Future studies involving both sexes are therefore warranted. In addition, the pharmacokinetic profile of SPM after intra-articular administration—including its retention, distribution, and clearance in the joint—has not been fully characterized. Although intra-articular injection can enhance local drug exposure, the concentration-time relationship of SPM in the joint space still requires quantitative assessment. Future studies should focus on determining the pharmacokinetic parameters and dose–response relationships of SPM, exploring new delivery strategies such as nanocarriers or molecular modifications to optimize its pharmacokinetic properties [26] and further validating long-term dosing regimens and biosafety. Although the si-NC used in the knockdown-efficiency experiments did not affect Odc1 expression, and exogenous SPM supplementation fully reversed the si-Odc1-induced phenotypes—evidence supporting the specificity of the observed effects—we acknowledge that including this control would further strengthen the conclusions, and it will be incorporated in future studies.

In summary, this study explored the therapeutic potential and underlying mechanisms of SPM in OA. Our data show that SPM alleviates OA by downregulating senescence-associated factors and ECM-degrading enzymes, upregulating matrix anabolic factors, and suppressing the activation of the NF-κB signaling pathway. Furthermore, *Odc1* was identified as a key regulator of endogenous SPM synthesis. Collectively, these findings suggest that SPM is a promising candidate for OA therapy that deserves further investigation.

## 4. Materials and Methods

### 4.1. Network Pharmacology Analysis

To identify the potential targets and molecular mechanisms underlying the therapeutic effect of SPM on OA, we retrieved candidate SPM targets from SwissTargetPrediction and PubChem and obtained OA-associated targets from GeneCards and OMIM using “osteoarthritis” as the search keyword. Overlapping targets from the two datasets were defined as potential therapeutic targets. Next, we constructed a protein–protein interaction (PPI) network for Homo sapiens via the STRING database, with a minimum interaction score set at 0.4, and identified hub targets ranked by degree using Cytoscape (version 3.9.1).

### 4.2. Chondrocyte Isolation and Culture

Primary chondrocytes were isolated from the knee cartilage of 8-week-old male Wistar rats. Rats were euthanized via cervical dislocation under deep isoflurane anesthesia, and the cartilage was minced into approximately 1 mm^3^ fragments. The fragments were next digested with 0.2% type II collagenase (Wuhan Servicebio Technology Co., Ltd., Wuhan, China) at 37 °C in a 5% CO_2_ incubator for 12–16 h. Isolated cells were cultured in DMEM/F12 medium (Wuhan Servicebio Technology Co., Ltd.) supplemented with 10% FBS (Gibco; Thermo Fisher Scientific, Inc., Waltham, MA, USA) and 1% penicillin–streptomycin, incubated at 37 °C in a humidified 5% CO_2_ atmosphere, with the culture medium renewed every 2–3 days. Chondrocytes at passages 1 to 3 were used for all experiments. To establish the in vitro OA model, cells were treated with 40 µM TBHP (cat. no. B776231; Shanghai Macklin Biochemical Co., Ltd., Shanghai, China) for 24 h. Untreated cells served as the negative control, while TBHP-treated cells were used as the model control.

### 4.3. Enzyme-Linked Immunosorbent Assay (ELISA)

SPM concentrations in cell lysates were measured with a competitive SPM ELISA kit (cat. no. EU2592; Wuhan Fine Biotech Co., Ltd., Wuhan, China) following the manufacturer’s instructions. Briefly, 50 µL of cell lysate or standard solution was added to each well of a 48-well microplate pre-coated with SPM antigen, followed immediately by 50 µL of biotin-labeled detection antibody. The plate was gently mixed and incubated for 45 min at 37 °C. After incubation, the wells were washed five times with wash buffer. Next, 100 µL of horseradish peroxidase (HRP)-conjugated streptavidin was added to each well, followed by another 30 min of incubation at 37 °C. After a second washing step, 100 µL of 3,3′,5,5′-tetramethylbenzidine (TMB) substrate solution was added, and the plate was incubated for 15 min at 37 °C in the dark. The enzymatic reaction was stopped by adding 50 µL of stop solution, and the optical density (OD) was measured at 450 nm with a microplate reader. To normalize for variations in total protein content across different samples, the measured SPM level of each sample was divided by the total protein concentration of the corresponding cell lysate, which was determined using a BCA protein assay kit. Final SPM concentrations are expressed as ng/mL.

### 4.4. Senescence-Associated β-Galactosidase (SA-β-Gal) Staining

SA-β-Gal activity in chondrocytes was assessed using an SA-β-Gal staining kit (cat. no. G1073-100T; Wuhan Servicebio Technology Co., Ltd.) according to the manufacturer’s instructions. Briefly, cells were washed three times with PBS, fixed with fixation solution for 15 min at room temperature, and then rewashed with PBS. Subsequently, the cells were incubated with SA-β-Gal staining working solution at 37 °C for 14 h. After incubation, images were captured under an optical microscope (Olympus, Tokyo, Japan), and the proportion of SA-β-Gal-positive cells was determined by counting five randomly selected fields per well.

### 4.5. Cell Counting Kit-8 (CCK-8) Assay

The effect of SPM on chondrocyte viability was evaluated using the Cell Counting Kit-8 (CCK-8; Wuhan Servicebio Technology Co., Ltd.). Chondrocytes were seeded in 96-well plates at a density of 5 × 10^3^ cells/well. After attachment, cells were treated with SPM at final concentrations of 0, 5, 10, 20, 40, 80, and 160 µM. At 24, 48, and 72 h post-treatment, 10 µL of CCK-8 solution was added to each well, and the plates were incubated at 37 °C for 2 h. The absorbance was then measured at 450 nm using a microplate reader (EnVision; PerkinElmer, Inc., Waltham, MA, USA). All experiments were performed in triplicate wells and repeated independently three times.

### 4.6. Reverse Transcription-Quantitative PCR (RT-qPCR)

Chondrocytes were seeded in 6-well plates at a density of 3 × 10^5^ cells/well and cultured for 24 h. Total RNA was extracted using an RNA isolation kit (cat. no. R0026; Beyotime Biotechnology, Shanghai, China) and RNA purity and concentration were measured with a microspectrophotometer. In a 20 µL reaction system, cDNA was synthesized from total RNA using a double-stranded cDNA synthesis kit (cat. no. G3330; Wuhan Servicebio Technology Co., Ltd.). Subsequently, qPCR was performed using 2× Universal SYBR Green fast qPCR Mix (cat. no. G3320; Wuhan Servicebio Technology Co., Ltd.) on a LightCycler^®^ 480 Real-Time PCR System (software version 1.5; Roche, Basel, Switzerland), with the following thermal cycling conditions: 95 °C for 3 min, followed by 40 cycles at 95 °C for 10 s, 60 °C for 30 s, and 72 °C for 30 s. GAPDH served as the internal control, and relative gene expression was calculated using the 2−ΔΔCq method [27]. Primer sequences for all target genes are listed in Table 1.

### 4.7. Western Blot

Total protein was extracted from chondrocytes using RIPA lysis buffer (cat. no. G2002; Wuhan Servicebio Technology Co., Ltd.) supplemented with the serine protease inhibitor phenylmethylsulfonyl fluoride (PMSF) (cat. no. G2007; Wuhan Servicebio Technology Co., Ltd.). Samples were lysed on ice for 30 min and then centrifuged at 12,000× *g* for 10 min at 4 °C. The supernatants were collected, and protein concentrations were measured with a BCA protein assay kit (cat. no. G2026; Wuhan Servicebio Technology Co., Ltd.). Protein samples were mixed with SDS loading buffer, heated at 99 °C for 10 min, and separated via 10% SDS-PAGE (50 μg protein per lane) at a constant voltage: 80 V for the stacking gel and 120 V for the resolving gel. The separated proteins were then transferred to polyvinylidene fluoride (PVDF) membranes (pre-activated with methanol) using a wet transfer system, run at 300 mA for 90 min on ice. After blocking with 5% non-fat milk diluted in TBST for 1 h at room temperature, the membranes were washed three times with TBST (10 min per wash) and then incubated overnight at 4 °C with the following primary antibodies: ODC1 (1:1000; cat. no. A3898; Abclonal Biotech Co., Ltd., Wuhan, China), COX-2 (1:1000; cat. no. A1253; Abclonal Biotech Co., Ltd.), iNOS (1:1000; cat. no. A14031; Abclonal Biotech Co., Ltd.), MMP3 (1:1000; cat. no. A22328; Abclonal Biotech Co., Ltd.), MMP13 (1:1000; cat. no. A1606; Abclonal Biotech Co., Ltd.), ADAMTS5 (1:1000; cat. no. A2836; Abclonal Biotech Co., Ltd.), IKKβ (1:1000; cat. no. 8943; Cell Signaling Technology, Inc. Danvers, MA, USA), p-IKKβ (1:1000; cat. no. 2697; Cell Signaling Technology, Inc.), IκBα (1:1000; cat. no. T55026; Abmart Shanghai Co., Ltd., Shanghai, China), p-IκBα (1:1000; cat. no. TP56280; Abmart Shanghai Co., Ltd.), p65 (1:1000; cat. no. AT55034; Abmart Shanghai Co., Ltd.), p-p65 (1:1000; cat. no. TP56372; Abmart Shanghai Co., Ltd., Shanghai, China), and GAPDH (1:4000; cat. no. GB15004; Wuhan Servicebio Technology Co., Ltd.). After three 10 min washes with TBST, the membranes were incubated with HRP-conjugated goat anti-rabbit secondary antibody (1:3000; cat. no. GB23303; Wuhan Servicebio Technology Co., Ltd.) for 1 h at room temperature. Following three more 10 min TBST washes, immunoreactive signals were developed with enhanced chemiluminescence (ECL) reagent (cat. no. G2020; Wuhan Servicebio Technology Co., Ltd.) and captured using the ChemiDoc Touch imaging system (Bio-Rad Laboratories, Inc., Hercules, CA, USA). Band intensities were quantified with ImageJ software (version 1.8; National Institutes of Health, Bethesda, MD, USA) and normalized to GAPDH, which served as the loading control.

### 4.8. Immunofluorescence (IF)

Chondrocytes at passages 1 to 3 were seeded onto sterile glass coverslips and cultured for 24 h. Cells were then fixed with 4% paraformaldehyde at room temperature for 30 min, washed three times with PBS, and permeabilized with 0.1% Triton X-100 in PBS for 10 min. After three additional PBS washes, cells were blocked with 1% bovine serum albumin (BSA) in PBS for 1 h at room temperature. After removing the blocking solution, cells were incubated overnight at 4 °C with primary antibodies against MMP3 (1:1000; cat. no. A22328; Abclonal Biotech Co., Ltd.), MMP13 (1:1000; cat. no. A1606; Abclonal Biotech Co., Ltd.), ADAMTS5 (1:1000; cat. no. A2836; Abclonal Biotech Co., Ltd.), aggrecan (1:500; cat. no. GB11373; Wuhan Servicebio Technology Co., Ltd.), and type II collagen (1:200; cat. no. GB11021; Wuhan Servicebio Technology Co., Ltd.). After washing with PBS, the cells were incubated with fluorescence-conjugated goat anti-rabbit IgG secondary antibody (1:400; ab150090; Abcam plc) for 1 h at room temperature in the dark. Nuclei were counterstained with 4′,6-diamidino-2-phenylindole (DAPI), and images were acquired using a confocal laser scanning microscope. Fluorescence intensity was quantified using ImageJ software (version 1.8).

### 4.9. Immunohistochemistry (IHC)

Paraffin-embedded sections were deparaffinized, rehydrated, and subjected to antigen retrieval in citrate buffer (pH 6.0). Non-specific binding was blocked with 5% BSA (cat. no. A8010; Beijing Solarbio Science & Technology Co., Ltd., Beijing, China) in PBS containing 0.5% Triton X-100 (PBST) for 30 min at room temperature. The sections were then incubated overnight at 4 °C with primary antibodies against ODC1 (1:200; cat. no. ab97395; Abcam plc), p16 (1:100; cat. no. WLH3673; Wanleibio Biotechnology Co., Ltd.), p21 (1:100; cat. no. WL01800; Wanleibio Biotechnology Co., Ltd., Shenyang, China), p65 (1:200; cat. no. T55034; Abmart Shanghai Co., Ltd.), and p-p65 (1:200; cat. no. TP56372; Abmart Shanghai Co., Ltd.). After three washes with PBS, the sections were incubated with HRP-conjugated goat anti-rabbit IgG secondary antibody (1:1000; cat. no. ab6721; Abcam plc) for 50 min at 37 °C. Immunoreactivity was visualized using a DAB substrate kit (cat. no. G1236; Wuhan Servicebio Technology Co., Ltd.), and nuclei were counterstained with hematoxylin. Images were captured using an inverted microscope (NIKON ECLIPSE CI, Tokyo, Japan), and quantitative analysis was conducted by measuring the integrated optical density using Image Pro Plus software (version 6.0).

### 4.10. Data Mining

To investigate *Odc1* expression in osteoarthritis (OA), we downloaded two human OA cartilage gene expression datasets, GSE114007 (RNA-seq, including 38 human knee cartilage samples) and GSE169077 (Affymetrix microarray, including 11 samples), from the Gene Expression Omnibus (GEO). All analyses were conducted using the processed expression matrices provided by GEO. The two matrices were merged by gene symbol, resulting in 11,707 shared genes across 49 samples. Since the two datasets were generated on different platforms (RNA-seq and microarray), we corrected batch effects using the ComBat function from the sva R package (version 3.60.0), with batches defined by dataset source. The correction effect was verified via principal component analysis (PCA). We identified differentially expressed genes (DEGs) between the OA group and the control group using the limma package with empirical Bayes moderation, with the screening threshold set at |log_2_FC| > log_2_(1.5) and adjusted *p* < 0.05 (Benjamini–Hochberg method). We then extracted *Odc1* expression data and compared its levels between the two groups. All statistical analyses and data visualization, including the generation of heatmaps and volcano plots, were performed in R (version 4.1.0) using the ggplot2 (version 3.3.5) and pheatmap (version 1.0.12) packages.

### 4.11. Odc1 Gene Silencing

Specific small interfering RNA targeting rat *Odc1* (si-Odc1) and negative control siRNA (si-NC) were synthesized by Beijing Tsingke Biotech Co., Ltd. Beijing, China, When chondrocytes reached 60–70% confluence, they were transfected with 50 nM siRNA using Lipofectamine 3000 transfection reagent (Invitrogen, Carlsbad, CA, USA) according to the manufacturer’s instructions. Knockdown efficiency was assessed 48 h post-transfection by RT-qPCR and Western blot analysis of Odc1 mRNA and protein levels. in si-Odc1-transfected, si-NC-transfected, and untransfected cells. The target sequence of si-Odc1 was 5′-AGCAAGUGUCUCAGAUCAA-3′. All subsequent functional experiments were performed 48 h after transfection to ensure adequate gene silencing. In the functional assays performed under TBHP stimulation, si-Odc1-transfected cells were compared with TBHP-stimulated untransfected cells; si-NC-transfected cells served as the transfection control in the knockdown-efficiency experiments.

### 4.12. Histopathological Analysis

Rat knee joints were dissected and fixed in 4% paraformaldehyde at room temperature (20 °C) for 48 h. After fixation, the specimens were decalcified in decalcifying solution for 28 days to completely remove calcium. The decalcified joints were then dehydrated through a graded ethanol series, embedded in paraffin, and coronally sectioned at a thickness of 4–5 µm. The sections were subsequently processed for the following staining procedures. For hematoxylin and eosin (H&E) staining, sections were stained with hematoxylin for 5 min and eosin for 2 min to evaluate cartilage structural integrity, chondrocyte morphology, and synovial inflammation. For Safranin O-Fast Green staining, sections were stained with Fast Green for 2 min, followed by Safranin O for 3 min, to assess proteoglycan content and distribution within the cartilage matrix. After staining, sections were dehydrated through a graded ethanol series, cleared in xylene, and mounted with neutral balsam. Images were captured using an upright fluorescence microscope (BX51; Olympus Corporation, Tokyo, Japan). Cartilage damage was semi-quantitatively evaluated using the Osteoarthritis Research Society International (OARSI) scoring system [28], with the following criteria: 0, intact cartilage surface; 1, surface fibrillation; 2, surface discontinuity; 3, vertical fissures; 4, cartilage erosion; 5, full-thickness cartilage loss; and 6, joint deformation.

### 4.13. Animal Experiments

All animal procedures were approved by the Animal Ethics Committee of Renmin Hospital of Wuhan University and were performed in strict accordance with the Guide for the Care and Use of Laboratory Animals (8th edition) published by the National Research Council [29]. This approval covered the use of 8-week-old rats for the in vivo model and 8-week-old rats for primary chondrocyte isolation.

A total of fifteen 8-week-old specific pathogen-free (SPF) male Wistar rats, weighing 240–280 g, were obtained from the Hubei Provincial Center for Disease Control and Prevention (Wuhan, China). Animals were housed under controlled conditions at 22 ± 2 °C with 50–60% humidity, maintained on a 12 h light/dark cycle, and provided with food and water ad libitum. After one week of acclimatization, the rats were randomly assigned to three groups (*n* = 5 per group) using a random number table: sham-operated (sham), anterior cruciate ligament transection (ACLT), and ACLT + SPM. The sample size (*n* = 5 per group) was determined based on a power analysis using G*Power software (version 3.1.9.7) (α = 0.05, power = 0.80), assuming a 30% difference in the primary outcome based on our preliminary experiments.

OA was induced by ACLT surgery. Briefly, rats were anesthetized with isoflurane (3.5% for induction, 1.5% for maintenance) delivered in 100% oxygen at a flow rate of 1.0 L/min via a face mask. Anesthetic depth was continuously monitored by toe-pinch reflex and respiratory rate throughout the procedure. The knee joint was then exposed. In the ACLT and ACLT + SPM groups, the anterior cruciate ligament was transected, while the sham group underwent joint capsule incision without ligament transection. For postoperative analgesia, rats received subcutaneous injections of buprenorphine (0.05 mg/kg) immediately after surgery and every 12 h for 48 h postoperatively.

All animals were monitored daily for clinical signs of distress, pain, or significant morbidity throughout the experimental period. The study established predefined humane endpoints to minimize suffering: (i) a >20% weight loss from baseline sustained for two consecutive days; (ii) complete loss of weight-bearing function on the affected limb or inability to access food and water; (iii) severe and persistent joint inflammation accompanied by ulceration or infection that did not respond to treatment; (iv) signs of respiratory distress; (v) persistent hypothermia or bradycardia; and (vi) a moribund state. None of the 15 rats reached any of these endpoints; all animals were euthanized at the predetermined time points in accordance with the approved animal care protocol.

Starting at one week post-surgery, rats in the ACLT + SPM group received a weekly intra-articular injection of 15 µL of sterile saline containing 10 µg SPM into the right knee joint. Rats in the sham and ACLT groups received an equal volume of sterile saline as controls. At the end of week 8, all rats were euthanized by cervical dislocation under deep anesthesia induced with 5% isoflurane (confirmed by the absence of pedal reflex), consistent with the American Veterinary Medical Association guidelines [30], and knee joint specimens were harvested for subsequent analyses.

### 4.14. Statistical Analysis

All experiments were independently repeated at least three times, with each repetition counted as an independent biological replicate. The Shapiro–Wilk test was used to assess the normality of data distribution, while Levene’s test was applied to evaluate variance homogeneity. Normally distributed data are expressed as mean ± standard deviation (SD) and analyzed with parametric tests: Student’s *t*-test (for equal variances) or Welch’s *t*-test (for unequal variances) for two-group comparisons and one-way analysis of variance (ANOVA) with Tukey’s post hoc test for comparisons among multiple groups. Non-normally distributed data are presented as median (interquartile range) and analyzed with non-parametric tests: the Mann–Whitney U test for two-group comparisons and the Kruskal–Wallis test for multiple-group comparisons. All statistical analyses were conducted using GraphPad Prism 8.0 software. A *p*-value < 0.05 was defined as the threshold for statistical significance. All individual data points are displayed as scatter plots overlaid on the bar graphs in the corresponding figures.

## Figures and Tables

**Table 1:** Primer sequences used for reverse transcription-quantitative PCR analysis.

Gene Name	F/R	Sequences 5′-3′
*ODC1*	F	CAGAGCCATAGTGAGCACCC
	R	ATCCGCAAAACCAACTTTGCC
*p16*	F	CGCAGGTTCTTGGTCACTGT
	R	TGTTCACGAAAGCCAGAGCG
*p21*	F	CCTGGTGATGTCCGACCTG
	R	CCATGAGCGCATCGCAATC
*MMP3*	F	GTCCAGAAGATCGATGATGACC
	R	TCCAACTGTGAAGATCCGCT
*MMP13*	F	GCCACCTTCTCTTTGTGTGTGAGTG
	R	GACTTCTTCAGGATTCCCCGCA
*ADAMTS5*	F	ACAAGAGTCTGGAGGTGAGCAAG
	R	ACATATGGTCCCAACGTCTGC
GAPDH	F	GCAAGTTCAACGGCACAG
	R	GCCAGTAGACTCCACGACAT

F: forward primer; R: reverse primer.

## Data Availability

The original contributions presented in this study are included in the article. Further inquiries can be directed to the corresponding author.

## Supplementary Materials

The following supporting information can be downloaded at https://www.mdpi.com/article/10.3390/ijms27219859/s1.

## References

[1] Peng X., Chen X., Zhang Y., Tian Z., Wang M., Chen Z. (2025). Advances in the pathology and treatment of osteoarthritis. J. Adv. Res..

[2] Long H., Liu Q., Yin H., Wang K., Diao N., Zhang Y., Lin J., Guo A. (2022). Prevalence Trends of Site-Specific Osteoarthritis from 1990 to 2019: Findings from the Global Burden of Disease Study 2019. Arthritis Rheumatol..

[3] Qiao L., Li M., Deng F., Wen X., Deng H., Xue Z., Zhou J., Lin J., Amhare A.F., Xiang R. (2025). Epidemiological trends of osteoarthritis at the global, regional, and national levels from 1990 to 2021 and projections to 2050. Arthritis Res. Ther..

[4] Chen B., Ning K., Sun M.L., Zhang X.A. (2023). Regulation and therapy, the role of JAK2/STAT3 signaling pathway in OA: A systematic review. Cell Commun. Signal..

[5] Cooper C., Chapurlat R., Al-Daghri N., Herrero-Beaumont G., Bruyère O., Rannou F., Roth R., Uebelhart D., Reginster J.-Y. (2019). Safety of Oral Non-Selective Non-Steroidal Anti-Inflammatory Drugs in Osteoarthritis: What Does the Literature Say?. Drugs Aging.

[6] Zhang Y., Han Y., Sun Y., Hao L., Gao Y., Ye J., Wang H., Zhang T., Liu Y., Yang Y. (2026). Osteoarthritis: Molecular pathogenesis and potential therapeutic options. Signal Transduct. Target. Ther..

[7] He R., Wei Y., Peng Z., Yang J., Zhou Z., Li A., Wu Y., Wang M., Li X., Zhao D. (2024). α-Ketoglutarate alleviates osteoarthritis by inhibiting ferroptosis via the ETV4/SLC7A11/GPX4 signaling pathway. Cell. Mol. Biol. Lett..

[8] Deng Y., Liu J., Bamahel A.S., Zhang P., Yu A., Cui H., Xu H., Fang Y. (2026). Chondrocyte inflammatory-immune responses in osteoarthritis: From pathogenesis to therapeutic intervention. Int. Immunopharmacol..

[9] Fu B., Shen J., Zou X., Sun N., Zhang Z., Liu Z., Zeng C., Liu H., Huang W. (2024). Matrix stiffening promotes chondrocyte senescence and the osteoarthritis development through downregulating HDAC3. Bone Res..

[10] Abbas M., Cruz C.J., Tamargo J.A., Lipat A.L., Fareed R., Deaton L., Gordon C., Johnson A., Sharma B., Leeuwenburgh C. (2025). Exploring senescence markers as potential drivers of osteoarthritis pain in aging adults. Exp. Gerontol..

[11] Yang J., Zheng W., Mei Y., Ni H., Liu H., Zhang Y., Li Y., Chen H., Cai L., Tian N. (2025). Eriocitrin Mitigates Osteoarthritis Development by Suppressing Chondrocyte Inflammation and ECM Degradation via Nrf2/HO-1/NF-κB Pathways. J. Agric. Food Chem..

[12] Dang Y., Liu Y., Zhang B., Zhang X. (2025). Aging microenvironment in osteoarthritis focusing on early-stage alterations and targeted therapies. Bone Res..

[13] Liu S., Mou J., Xiong Z., Liu J., Du C., Zhu Z., Tan J., Cheng S., Qiu X., Huang W. (2025). Interferon-γ-Induced Mesenchymal Stem Cell Exosomes Attenuate Chondrocyte Senescence and Osteoarthritis Progression by Hsp70. ACS Nano.

[14] Luo D., Lu X., Li H., Li Y., Wang Y., Jiang S., Li G., Xu Y., Wu K., Dou X. (2024). The Spermine Oxidase/Spermine Axis Coordinates ATG5-Mediated Autophagy to Orchestrate Renal Senescence and Fibrosis. Adv. Sci..

[15] Soda K. (2020). Spermine and gene methylation: A mechanism of lifespan extension induced by polyamine-rich diet. Amino Acids.

[16] Gutierrez G.E.J., Jiménez F.V.B., Muñoz L.G., Guerrero Y.S.T., Melo N.M.M., Cristóbal-Luna J.M., Garcia N.L., Cordero-Martínez J., Magaña J.J. (2023). The Molecular Role of Polyamines in Age-Related Diseases: An Update. Int. J. Mol. Sci..

[17] Tootsi K., Vilba K., Märtson A., Kals J., Paapstel K., Zilmer M. (2020). Metabolomic Signature of Amino Acids, Biogenic Amines and Lipids in Blood Serum of Patients with Severe Osteoarthritis. Metabolites.

[18] Lin H., Lv Y., Liu H., Lin Z., Zhu N., Huang B. (2025). Baicalein alleviates chronic acute stress-induced irritable bowel syndrome-like symptoms in rats via modulating the ODC1/NF-κB pathway and oxidative stress. Biochem. Biophys. Res. Commun..

[19] Vasilogiannakopoulou T., Begou O., Karali C.S., Efthymiou G., Roubelakis M., Gorgoulis V., Gkouskou K.K., Gika H., Eliopoulos A.G. (2025). Putrescine functions as a metabolic checkpoint in replication stress-induced senescence. Cell. Mol. Life Sci..

[20] Lu H., Jia C., Wu D., Jin H., Lin Z., Pan J., Li X., Wang W. (2021). Fibroblast growth factor 21 (FGF21) alleviates senescence, apoptosis, and extracellular matrix degradation in osteoarthritis via the SIRT1-mTOR signaling pathway. Cell Death Dis..

[21] Sun K., Zhang X., Hou L., Lu F., Liu H., Zheng Z., Guo Z., Xu J., Ruan Z., Hou Y. (2025). TRPM2-mediated feed-forward loop promotes chondrocyte damage in osteoarthritis via calcium-cGAS-STING-NF-κB pathway. J. Adv. Res..

[22] Jin F., Li X., Lee H.J., Lee C.J. (2020). Diclofenac Inhibits Phorbol Ester-Induced Gene Expression and Production of MUC5AC Mucin via Affecting Degradation of IkBα and Translocation of NF-kB p65 in NCI-H292 Cells. Biomol. Ther..

[23] Mahalingam S.S., Pandiyan P. (2024). Polyamines: Key Players in Immunometabolism and Immune Regulation. J. Cell. Immunol..

[24] Facchini A., Borzì R.M., Olivotto E., Platano D., Pagani S., Cetrullo S., Flamigni F. (2012). Role of polyamines in hypertrophy and terminal differentiation of osteoarthritic chondrocytes. Amino Acids.

[25] Yao Q., Wu X., Tao C., Gong W., Chen M., Qu M., Zhong Y., He T., Chen S., Xiao G. (2023). Osteoarthritis: Pathogenic signaling pathways and therapeutic targets. Signal Transduct. Target. Ther..

[26] Sun M., Ma B., Pan Z., Zhao Y., Tian L., Fan Y., Kong W., Wang J., Xu B., Ao Y. (2024). Targeted Therapy of Osteoarthritis via Intra-Articular Delivery of Lipid-Nanoparticle-Encapsulated Recombinant Human FGF18 mRNA. Adv. Healthc. Mater..

[27] Livak K.J., Schmittgen T.D. (2001). Analysis of relative gene expression data using real-time quantitative PCR and the 2(-Delta Delta C(T)) Method. Methods.

[28] Gerwin N., Bendele A.M., Glasson S., Carlson C. (2010). The OARSI histopathology initiative—Recommendations for histological assessments of osteoarthritis in the rat. Osteoarthr. Cartil..

[29] National Research Council Committee for the Update of the Guide for the Care and Use of Laboratory A (2011). The National Academies Collection: Reports funded by National Institutes of Health. Guide for the Care and Use of Laboratory Animals.

[30] AVMA Panel on Euthanasia, American Veterinary Medical Association (2001). 2000 Report of the AVMA Panel on Euthanasia. J. Am. Vet. Med. Assoc..

