# Supplementary material for: Spermine Alleviates Chondrocyte Senescence and Extracellular Matrix Degradation by Suppressing NF-κB Signaling, with Odc1 Acting as a Potential Upstream Regulator
Source: Int J Mol Sci. 2026 Nov 3;27(21):9859. doi: 10.3390/ijms27219859 (PMC13763568; doi:10.3390/ijms27219859)
Supplement: Supplementary file 1 [file ijms-27-09859-s001.zip › ijms-4567064-supplementary.pdf]

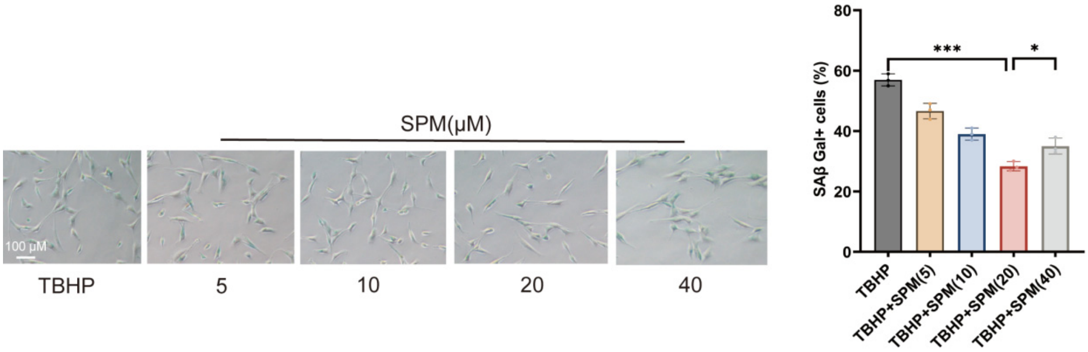

Figure S1. Dose-dependent effects of SPM on TBHP-induced chondrocyte senescence. Primary chondrocytes were treated with 40 μM TBHP for 24 h, with or without SPM at the indicated concentrations. Cellular senescence was assessed using SA-β-gal staining. Representative images are presented in the left panels, and the proportion of SA-β-gal-positive cells is quantified in the right panel. Data are expressed as mean ± SD (n = 3). **p* < 0.05, ****p* < 0.001 vs. the TBHP group. Scale bar: 100 μm.
